# Antidepressant prescribing pattern in Croatia: a retrospective, longitudinal study from 2017 to 2022

**DOI:** 10.3325/cmj.2026.67.55

**Published:** 2026-04

**Authors:** Katarina Gvozdanović, Roberto Mužić, Helena Orehovački, Danijela Štimac Grbić

**Affiliations:** 1 Department of Pharmacoepidemiology, Teaching Institute of Public Health, Zagreb, Croatia; 2 Croatian Institute of Public Health, Zagreb, Croatia; 3 Health Care Center Zagreb – Centar, Zagreb, Croatia; Gvozdanović et al: Antidepressant prescribing pattern in Croatia

## Abstract

**Aim:**

To assess antidepressant prescribing patterns in Croatia.

**Methods:**

This retrospective study assessed electronic prescription patterns for antidepressants (Anatomical Therapeutic Chemical code N06A) in Croatia from 2017 to 2022. We collected data on annual prescription counts, drug types, drug utilization, utilization trends, indications, and patient demographics.

**Results:**

Over six years, 8.66 million antidepressant prescriptions were issued to 415 328 patients in Croatian primary care. Utilization increased by 22.32% (from 22.4 to 27.4 defined daily doses per 1000 inhabitants per day) but remained below the European average. Selective serotonin reuptake inhibitors accounted for nearly 60% of prescriptions. Women and older adults (≥65 years) were the primary users.

**Conclusion:**

Despite rising antidepressant use, Croatia lags behind other European countries, possibly due to high benzodiazepine use. The prescribing pattern highlights the need for targeted, evidence-based mental health policies. Special attention should be given to pediatric patients, the elderly, and patients affected by war-related trauma, while also exploring socioeconomic influences on prescribing practices.

According to the World Health Organization (WHO), approximately 280 million people worldwide suffer from depression (1). Before 2020, depressive and anxiety disorders were among the leading causes of the global health-related burden, which was further affected by the COVID-19 pandemic and related lockdowns (2). The lockdowns exacerbated stress, anxiety, and depression in the population, but the complex impact of the COVID-19 pandemic on mental health trends is still not fully understood (3).

Antidepressants constitute the first-line treatment for depression (4). They are widely prescribed, and their use continues to increase globally, influenced by growing mental health awareness, expanded indications, demographic changes, updated clinical guidelines, and evolving medical practices (5,6). In the EU, their consumption is steadily increasing; however, specific trends vary by country (7-12).

Previous research has demonstrated a growing trend in antidepressant use in Croatia, although these medications are prescribed significantly less frequently than anxiolytics and are used at lower rates than in other EU countries (13,14). Antidepressants are generally prescribed by general practitioners in primary care settings, as well as by specialists in psychiatry and, in some cases, neurology. General practitioners can prescribe antidepressants independently for simpler cases of depression or anxiety. However, in more severe or complex cases, they typically prescribe antidepressants based on recommendations from a specialist. Pediatric psychiatrists may also prescribe these medications for children and adolescents.

There are 90 registered brand-name antidepressants in Croatia, 50 of which are included on the Basic Drug List and are fully covered by basic health insurance. Notably, the Croatian Institute for Health Insurance provides health coverage for over 95% of the population, as specified in the Law on Compulsory Health Insurance (15). Additionally, 20 antidepressants are listed on the Supplementary List, requiring a co-payment of up to 80% of the drug's price (16). Together, these arrangements ensure that patients have access to a wide range of therapeutic options tailored to their needs.

Previous research assessed prescribing practices in Croatia by predominantly relying on sales data. This is the first study to use the digital system of ePrescriptions to assess nationwide antidepressant prescribing practices in Croatia from 2017 to 2022.

## Methods

This longitudinal, retrospective study is based on the analysis of all electronic prescriptions for antidepressants prescribed in Croatia from January 1, 2017, to December 31, 2022. It follows the Strengthening the Reporting of Observational Studies in Epidemiology guidelines (17). The study covers a national sample from the primary care setting. The Anatomical-Therapeutic-Chemical (ATC) Classification code N06A was a criterion for prescription retrieval (18). Hospital use of antidepressants was not investigated. Data were sourced from the Croatian Institute of Public Health (CIPH). The Ethics Committee of CIPH granted a waiver due to secondary use of data and data anonymization.

We assessed both the number of prescriptions and estimated drug consumption. For each prescription, the total quantity of active substance and defined daily doses (DDDs) were calculated, considering the defined daily dose assigned by the WHO (ATC DDD) for each active substance. Consumption is expressed in DDDs per 1000 inhabitants per day (DID) (19). We assessed both the overall and individual use for every antidepressant. The number of inhabitants was obtained from the Croatian Bureau of Statistics (20).

Prescriptions were grouped by active substance (ATC7 level). Each prescription contains mandatory information on patient age and gender. Patients were grouped into age groups: 0-17, 18-25, 26-44, 45-65, and 65+ years. We separately analyzed data by gender, both in the total sample and in different age groups.

Each prescription contains an indication, coded according to the ICD 10 classification (up to one decimal point) (21). The frequency of individual indications was determined separately for each age group (the percentage was calculated in relation to the total number of prescriptions in each age group). Indications were not grouped (eg, F32, F32.1, and F32.2 were considered as separate indications). Only one indication can be coded within one prescription, so the recorded code may not fully capture all the underlying clinical conditions.

### Statistical analysis

Linear regression was used to identify trends in time series: *P* values, R-squared values, and trends (increase, decrease, or no trend). Spearman’s rank correlation coefficient was used to evaluate monotonic trends without assuming linear relationships. Statistical analysis and graph design were performed using Python 3.10.

## Results

**Figure 1:**
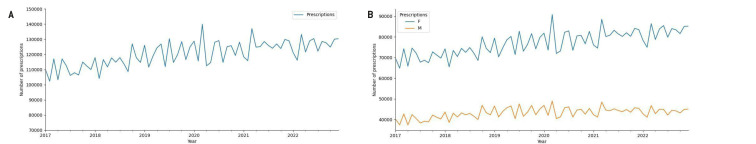
(**A**) The number of monthly prescriptions for antidepressants between January 1, 2017, and December 31, 2022, in a Croatian primary care setting. (**B**) The number of prescriptions according to gender.

We retrieved 8660.940 prescriptions for antidepressants prescribed to 415 328 individual patients over a period of 6 years in a Croatian primary care setting. The monthly number of prescriptions for antidepressants increased from 109 817 (January 2017) to 130 293 (December 2022) with the peak in March 2020 (139,792) (Figure 1A). Female patients were prescribed 64.1% of all prescriptions, with a consistent female/male ratio over the entire period (Figure 1B).

**Figure 2:**
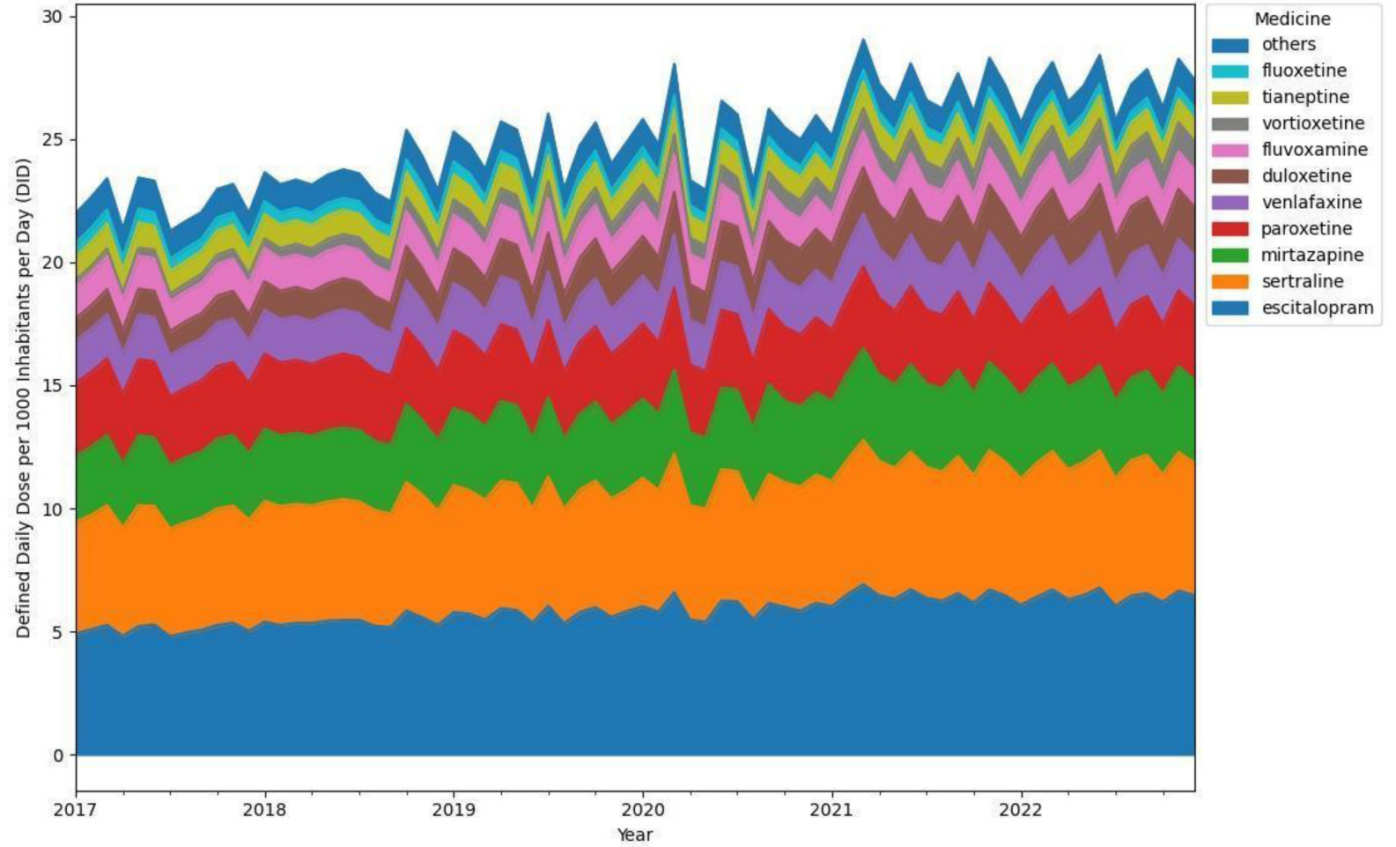
The consumption of antidepressants (in defined daily doses per 1000 inhabitants) in Croatian primary care setting from January 1, 2017 to December 31, 2022 by active substances. Ten active substances with the highest relative consumption are presented individually, while 8 substances with the lowest total consumption are grouped under the label “others.”

From 2017 to 2022, the utilization of antidepressants increased from 22.4 to 27.4 DID (Figure 2). Eighteen different active substances were present within 102 different drug packages prescribed. The most frequently consumed antidepressant was escitalopram, followed closely by sertraline, paroxetine, and mirtazapine. These four medications make up for almost 70% of total antidepressant consumption (Figure 2).

Tianeptine, citalopram, amitriptyline, fluoxetine, maprotiline, agomelatine, and moclobemide experienced a declining trend, while no trend was noted for paroxetine and reboxetine. The remaining substances (escitalopram, sertraline, mirtazapine, duloxetine, venlafaxine, fluvoxamine, vortioxetine, trazodone, and bupropion) exhibited an increasing trend (Supplemental Figure 1(Supplementary Figure 1)).

**Figure 3:**
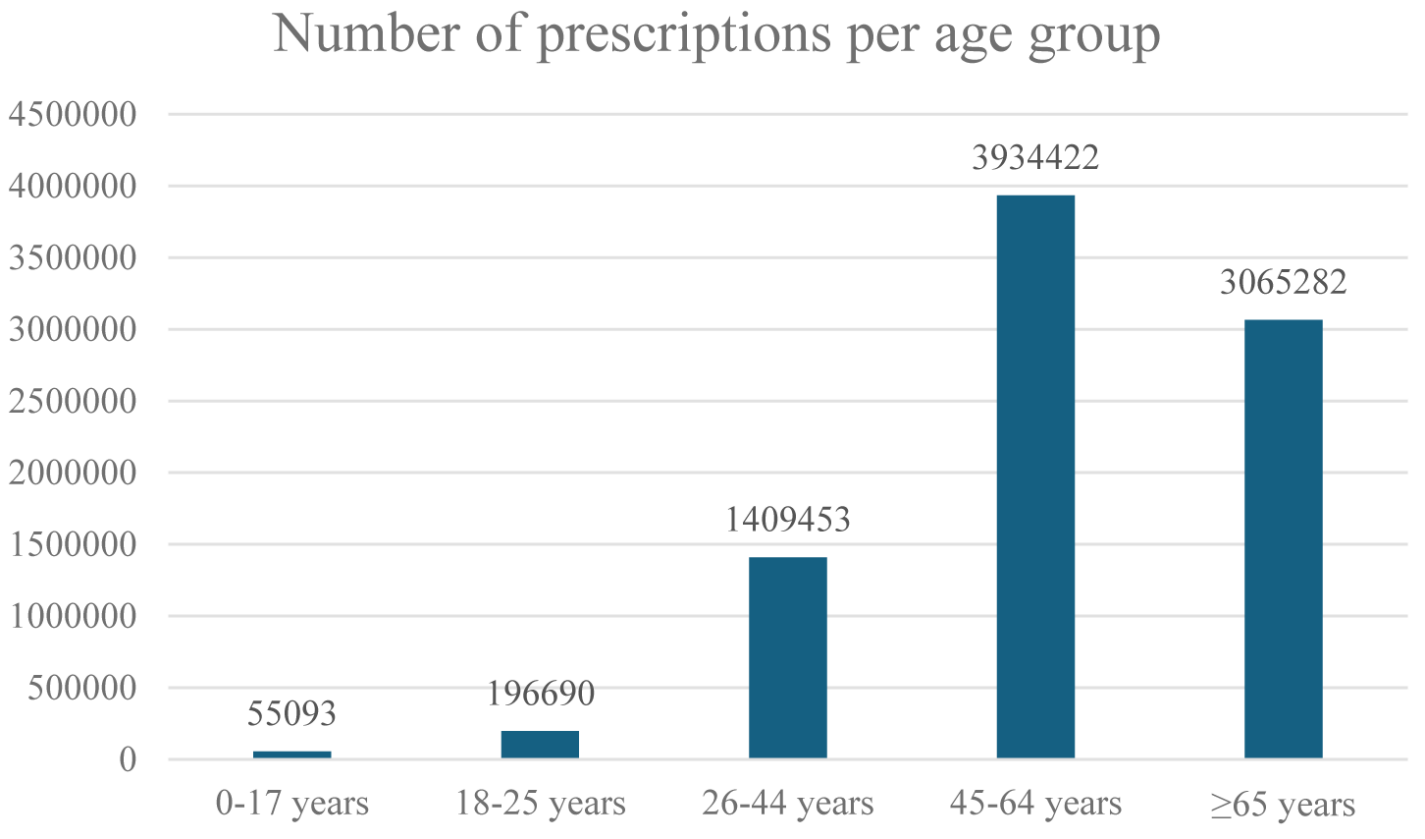
Prescription distribution across all age groups.

The highest number of prescriptions was in the age group 45-64 years (45.43% of all) (Figure 3, Table 1). When prescriptions were stratified jointly by age group and gender, women predominated in every stratum (Table 1). Population-adjusted prescribing rates ranged from 18.3/1000/y (women 0-17) to 740.4/1000/y (women 65+) (Supplemental Table 1(Supplementary Table 1)). After correcting for the gender composition of the Croatian population, the F/M ratio in the 65+ group decreased from 2.70 to 1.88, which indicates that approximately one-third of the apparent sex disparity in that group was attributable to the larger proportion of women in the elderly population. The highest adjusted F/M ratio was observed in the 0-17 group (2.13), while working-age groups showed a more uniform ratio of 1.38-1.62.

**Table 1:** Prescription data stratified by gender and age group. For each age group, total prescription counts and prescribing rates (per 1000 persons per year) are presented, together with data for the three antidepressants with the highest prescription counts during the reporting period*

Age group (years)	Gender	Total Rx (n)	% within age group	Rx/1000/y	Escitalopram (n)	Escitalopram (Rx/1000/y)	Sertraline (n)	Sertraline (Rx/1000/y)	Paroxetine (n)	Paroxetine (Rx/1000/y)
0-17	F	36 833	66.9	18.3	5661	2.8	18 449	9.2	263	0.1
	M	18 260	33.1	8.6	1734	0.8	9147	4.3	178	0.1
18-25	F	118 584	60.3	115	32 840	31.8	29 886	29	6313	6.1
	M	78 106	39.7	71	17 413	15.8	17 206	15.6	4203	3.8
26-44	F	833 203	59.1	289.5	238 246	82.8	169 387	58.9	75 411	26.2
	M	576 250	40.9	192.2	117 648	39.2	96 600	32.2	53 878	18
45-64	F	2 321 720	59	676.9	509 964	148.7	415 983	121.3	231 038	67.4
	M	1 612 702	41	491.2	264 929	80.7	288 410	87.8	144 169	43.9
65+	F	2 237 536	73	740.4	456 596	151.1	354 741	117.4	253 114	83.7
	M	827 746	27	393,3	154 965	73.6	129 691	61.6	72 637	34.5

*Abbreviations: F – females; M – males; Rx – prescriptions.

**Figure 4:**
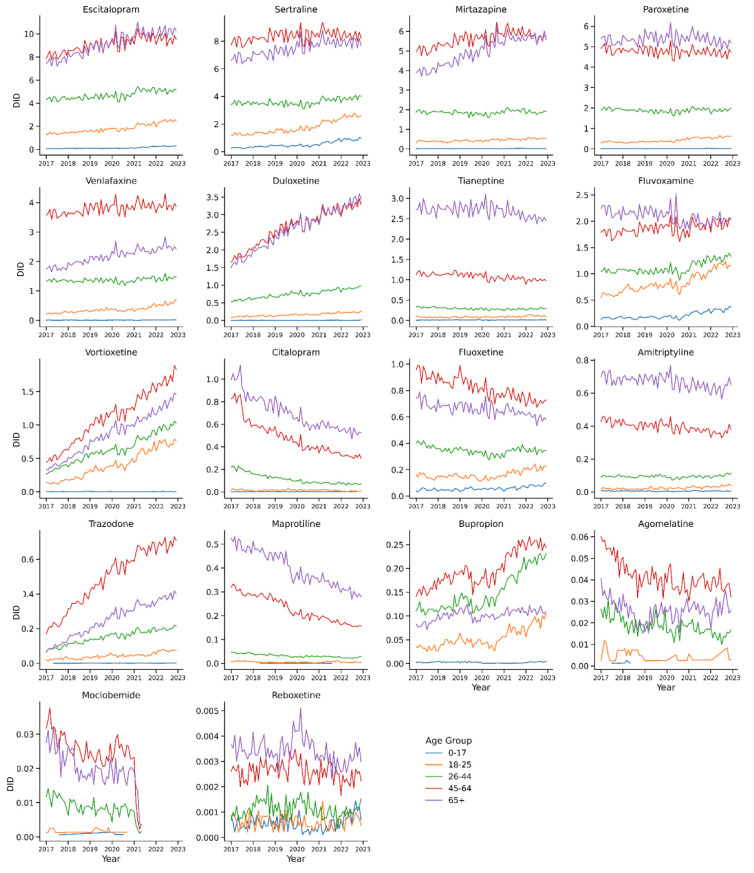
Monthly utilization of antidepressants between 2017 and 2022 stratified by age groups. The data are presented separately for every antidepressant (at the level of the active substance). DID – defined daily dose per 1000 inhabitants per day.

The highest utilization for individual antidepressants generally alternated between the age group 45-64 and the age group ≥65 years (Figure 4). The only exception was bupropion, which was most frequently consumed in the age group 26-44 years (Figure 4).

We also examined all prescriptions to investigate the indications for which the drug was prescribed. Every prescription can contain only one indication chosen by the physician at the time of prescribing. Table 2 shows the ten most frequent indications for every patient age group.

**Table 2:** Indications (ICD 10 code) retrieved from the prescriptions stratified by different age groups. Only the top 10 indications per age group are presented. The frequency of individual indications was determined for each age group separately (the percentage was calculated in relation to the total number of prescriptions in each age group). Indications were not grouped (eg, F32, F32.1, and F32.2 were considered as separate indications). Only one indication can be coded within the prescription

Patient age (years)	Total number of prescriptions per age group	Number of prescriptions per different ICD code	Percentage of prescriptions per different ICD code (%)	Indication for drug prescription (ICD-10 code)	Indication for drug prescription
0-17	55 093	6288	11.41	F93	Emotional disorders with onset specific to childhood
		5286	9.59	F93.8	Other childhood emotional disorders
		3682	6.68	F32	Depressive episode
		2581	4.68	F41.2	Mixed anxiety and depressive disorder
		2114	3.84	F43.2	Adjustment disorders
		1953	3.54	F41	Other anxiety disorders
		1846	3.35	F42	Obsessive-compulsive disorder
		1576	2.86	F32.1	Moderate depressive episode
		1521	2.76	F43.0	Acute stress reaction
		1507	2.74	F92	Mixed disorders of conduct and emotions
18-25	196 690	32 681	16.62	F41.2	Mixed anxiety and depressive disorder
		16 709	8.50	F41	Other anxiety disorders
		14 736	7.49	F41.0	Panic disorder (episodic paroxysmal anxiety)
		14 578	7.41	F32	Depressive episode
		9475	4.82	F43.2	Adjustment disorders
		7201	3.66	F41.1	Generalized anxiety disorder
		6180	3.14	F32.1	Moderate depressive episode
		5563	2.83	F32.2	Severe depressive episode without psychotic symptoms
		4512	2.29	F42	Obsessive-compulsive disorder
		4213	2.14	F60.3	Emotionally unstable personality disorder
26-44	1 409 453	232 841	16.52	F41.2	Mixed anxiety and depressive disorder
		147 126	10.44	F32	Depressive episode
		111 305	7.90	F41	Other anxiety disorders
		81 434	5.78	F41.0	Panic disorder (episodic paroxysmal anxiety)
		59 232	4.20	F43.2	Adjustment disorders
		56 080	3.98	F41.1	Generalized anxiety disorder
		38 707	2.75	F32.1	Moderate depressive episode
		37 089	2.63	F11.2	Mental and behavioral disorders due to use of opioids
		35 575	2.52	F32.2	Severe depressive episode without psychotic symptoms
		33 058	2.35	F33.2	Recurrent depressive disorder, current episode severe without psychotic symptoms
45-64	3 934 422	546 647	13.89	F32	Depressive episode
		496 460	12.62	F41.2	Mixed anxiety and depressive disorder
		401 694	10.21	F43.1	Posttraumatic stress disorder
		237 663	6.04	F33.2	Recurrent depressive disorder, current episode severe without psychotic symptoms
		192 519	4.89	F41	Other anxiety disorders
		154 431	3.93	F32.2	Severe depressive episode without psychotic symptoms
		134 449	3.42	F33	Recurrent depressive disorder
		133 118	3.38	F43.2	Adjustment disorders
		129 219	3.28	F32.1	Moderate depressive episode
		95 661	2.43	F33.1	Recurrent depressive disorder, current episode moderate
≥65	3 065 282	736 659	24.03	F32	Depressive episode
		329 819	10.76	F41.2	Mixed anxiety and depressive disorder
		164 809	5.38	F41	Other anxiety disorders
		141 545	4.62	F33	Recurrent depressive disorder
		132 946	4.34	F06.3	Organic mood [affective] disorders
		119 845	3.91	F32.1	Moderate depressive episode
		114 890	3.75	F33.2	Recurrent depressive disorder, current episode severe without psychotic symptoms
		98 659	3.22	F32.2	Severe depressive episode without psychotic symptoms
		63 207	2.00	F33.1	Recurrent depressive disorder, current episode moderate
		62226	2.03	F32.0	Mild depressive episode

## Discussion

In this study, the use of antidepressants in Croatia showed a steady increase from 22.4 DID in 2017 to 27.4 DID in 2022 (a 22.32% increase). Comparable upward trends have been noted internationally. For example, Slovenia, a neighboring country with a similar health care system, recorded a 38% increase in antidepressant use between 2009 and 2018 (12). In England, prescriptions for the 10 most commonly used antidepressants rose by 25% from 2015 to 2019 (22), while Australia and the Netherlands reported increases of 40% and 10%, respectively, over a comparable period (23).

Despite the increase, Croatia's antidepressant utilization (even when we add antidepressant hospital utilization) remains lower than the European average according to the Organisation for Economic Co-operation and Development (37 DDD per 1000 people per day in 2023) (24,25). This discrepancy may be attributed to Croatia's higher reliance on benzodiazepines compared with some EU countries (26). This issue needs to be addressed by health administration because benzodiazepines do not treat the underlying causes of depression or anxiety and may provide only short-term symptom relief, potentially delaying appropriate treatment and worsening long-term outcomes.

Our study did not specifically investigate the impact of the COVID-19 pandemic on antidepressant consumption; however, certain utilization patterns noticed during the study period are likely influenced by the pandemic. For instance, the peak in the number of prescriptions and overall consumption in March 2020 may reflect the stockpiling behavior at the beginning of the pandemic. While this finding should be interpreted with caution, a similar rationale has been proposed to explain antidepressant utilization patterns during the pandemic in Scandinavia, France, and Northern Ireland (26-28).

Similarly, the spike in fluvoxamine consumption during this period (especially in older age groups) could be linked to fluvoxamine’s potential ability to reduce the risk of hospitalization for patients with COVID-19 (29). Although our data show an increased antidepressant consumption during the COVID-19 pandemic in Croatia, this trend cannot be simply attributed to the pandemic, but neither can its potential influence be ignored. Previous research conducted in Croatia has reported no significant impact of the COVID-19 pandemic on overall antidepressant prescribing trends (30). A recent EU cross-national study using interrupted time series methodology suggested that while the pandemic did not substantially alter long-term utilization trends across European countries including Croatia, it might have resulted in short-term fluctuations and subgroup-specific effects (25). Our results are consistent with this interpretation, as we observed a temporary increase in prescribing. Differences between studies may be partly explained by variations in data sources and analytical approaches, including the use of aggregated sales data vs individual-level prescription data.

The most commonly prescribed antidepressants in Croatia were selective serotonin reuptake inhibitors (SSRIs) (escitalopram, sertraline, paroxetine, fluoxetine, fluvoxamine, and citalopram), accounting for 60% of total antidepressant utilization at the end of 2022. This trend is consistent with comparable studies from other European countries. In Slovenia, SSRIs accounted for 70% of all antidepressant prescriptions, with escitalopram and sertraline being the most frequently prescribed (12). In Denmark, SSRIs made up 48.5% of the 4.84 million antidepressant prescriptions issued to older adults between 2015 and 2019 (31). These trends align with clinical guidelines, which recommend SSRIs for their effectiveness and tolerability (4). The use of citalopram and fluoxetine exhibited a declining trend in our study, possibly as a result of the clinicians’ preference for escitalopram (32).

The use of tricyclic antidepressants amitriptyline and maprotiline showed a declining trend, likely due to their side effects, while moclobemide, a monoamine oxidase inhibitor, was discontinued from the market in June 2021. We observed a declining trend in tianeptine utilization, probably due to a shift in therapeutic preference to SSRIs, together with regulatory attention to its safety profile and reports of misuse, dependence, and withdrawal symptoms (33,34). The upward trend in bupropion use (among the 26-44 age group) may be attributed to its better tolerability compared with SSRIs and its lack of side effects such as weight gain and sexual dysfunction, which can be particularly important for individuals in this age group (35).

Antidepressant use by age typically peaks in the 45-64 and ≥65 age groups. In Slovenia, the most significant increase was observed among the elderly (over 80 years old), where antidepressant use surged by 25%, resulting in one in four individuals in this age group being prescribed antidepressants by 2018 (12). The rising trend in antidepressant utilization, especially among the elderly, underscores the need for targeted mental health interventions for this demographic. Policymakers should enhance support for mental health services and ensure the safe use of antidepressants. Also, future research in Croatia should examine the relationship between social deprivation and antidepressant prescribing patterns.

Indications noted in the prescriptions were generally consistent with expectations: among top ten indications for all age groups were mixed anxiety and depressive disorder, moderate depressive episode, and depressive disorder. A severe depressive episode without psychotic symptoms was present in all groups except for the age group 0-17 years, while adjustment disorder was present in all age groups except for patients ≥65 years.

Several findings deserve to be highlighted: the use of antidepressants for opioid dependence syndrome, PTSD treatment, and in the pediatric population. In addition to primary depressive and anxiety disorders, certain other diagnostic categories recorded in the prescriptions reflect the presence of complex psychiatric comorbidities that may influence antidepressant prescribing patterns. For instance, in patients diagnosed with opioid dependence syndrome (F11.2), antidepressants may be prescribed to address comorbid depressive or anxiety symptoms. In clinical practice, such treatment often occurs alongside opioid substitution therapy, most commonly with methadone or buprenorphine, which represents the standard pharmacological approach for opioid use disorder. The co-prescribing of antidepressants in this context reflects the need to manage complex psychiatric comorbidities that may affect treatment adherence and overall recovery outcomes. From a prescribing quality perspective, appropriate recognition and treatment of comorbid depression in patients receiving opioid substitution therapy is particularly important, as untreated depressive symptoms may negatively impact engagement with addiction treatment and increase the risk of relapse.

Pediatric prescribing warrants particular consideration: the increasing utilization trend is in line with other national studies, although overall prescribing levels remain within the lower range reported in Europe (36,37). We noticed a sex difference in antidepressant utilization in this age group (F/M ratio was 2.13) that mirrors wider sex-specific Croatian and European trends (36,38). Interestingly, the leading indication for children and adolescents is F93 (emotional disorders specific to childhood) rather than depression *per se.* Guidelines recommend pharmacological treatment only for moderate to severe depression, usually combined with psychotherapy, with fluoxetine (from age 8) as the preferred first-line SSRI due to the strongest evidence for efficacy and safety (39-41). In the EU, only fluoxetine, sertraline, and fluvoxamine have pediatric indications – fluoxetine for depression and the latter two for obsessive-compulsive disorder (42-44). The proportion of pediatric SSRI prescribing in our data set likely represents off-label use. While some health care professionals consider such prescribing clinically justified, the finding highlights the importance of adhering to guidelines, careful monitoring, and evaluating both efficacy and safety in young patients.

Posttraumatic stress disorder was the indication identified in over 400 000 prescriptions, occurring predominantly in the 45-64 age group. This distribution likely reflects the demographic profile of war veterans from the 1990s conflicts in Croatia. However, PTSD-related prescribing is likely underestimated as only one indication can be coded in a prescription: in routine clinical care, patients with PTSD are frequently coded under depressive disorders (F32 or F33) rather than PTSD (F43.1). This occurs because PTSD is commonly accompanied by significant depressive symptomatology, and comorbidity between PTSD and major depressive disorder is well documented. Depressive symptoms such as persistent low mood, anhedonia, sleep disturbances, and cognitive impairment may dominate the clinical presentation and therefore guide diagnostic coding and treatment decisions. As a result, antidepressants may be prescribed primarily for depressive symptom clusters, even when PTSD represents the underlying or comorbid condition.

Our study results highlight the need for continuous prescriber education, consistent classification criteria, and improved prescribing practices that prioritize evidence-based treatments for depression and anxiety. Additionally, targeted mental health interventions, especially for pediatric patients, the elderly, and underserved populations, could address gaps in care and align antidepressant utilization with European standards.

A limitation of this study is that utilization was calculated based on prescription data, which may not accurately reflect the actual medication use. Based on the unpublished information retrieved from the Croatian Health Insurance Fund, the dispensation of prescriptions is over 98%. Also, the study did not account for the so-called “private prescriptions.” Antidepressants in Croatia are dispensed at pharmacies with a valid prescription, and the majority of these prescriptions are reimbursed by the public health insurance fund. However, patients may also obtain antidepressants through private prescriptions, which are then stored in paper form in the pharmacy. The percentage of private prescriptions among all prescriptions is unknown. To the best of our knowledge, the percentage of private prescriptions among antidepressants may account for up to 10%, meaning that patterns of antidepressant use captured here can still adequately reflect clinical practice. Another limitation is the fact that only one diagnosis according to the ICD-10 code is recorded on prescriptions. This study could not consider the comorbidities that are widely present in psychiatric patients, and some diagnoses, such as PTSD, may have been underestimated. Although our study included all available prescription data, the trend analysis did not explicitly account for seasonal variation. As prescription patterns can fluctuate throughout the year, this may represent a potential source of confounding.

In conclusion, despite rising antidepressant use, Croatia lags behind other European countries, possibly due to high benzodiazepine use. The prescribing pattern highlights the need for targeted, evidence-based mental health policies. Special attention should be given to the pediatric patients, the elderly, and patients affected by war-related trauma, while also exploring socioeconomic influences on prescribing practices.

## References

[1] WHO. Depression. Available from: https://www.who.int/health-topics/depression*.* Accessed: March 19, 2026.

[2] COVID-19 Mental Disorders Collaborators Global prevalence and burden of depressive and anxiety disorders in 204 countries and territories in 2020 due to the COVID-19 pandemic. Lancet 2021 398 1700 12 10.1016/S0140-6736(21)02143-7 34634250 PMC8500697

[3] Holmes EA O’Connor RC Perry VH Tracey I Wessely S Arseneault L Multidisciplinary research priorities for the COVID-19 pandemic: a call for action for mental health science. Lancet Psychiatry 2020 7 547 60 10.1016/S2215-0366(20)30168-1 32304649 PMC7159850

[4] Overview | Depression in adults: treatment and management | Guidance | NICE. NICE; 2022. Available from: https://www.nice.org.uk/guidance/ng222. Accessed: March 19, 2026.

[5] Alabaku O Yang A Tharmarajah S Suda K Vigod S Tadrous M Global trends in antidepressant, atypical antipsychotic, and benzodiazepine use: A cross-sectional analysis of 64 countries. PLoS One 2023 18 e0284389 10.1371/journal.pone.0284389 37099524 PMC10132527

[6] Brauer R Alfageh B Blais JE Chan EW Chui CSL Hayes JF Psychotropic medicine consumption in 65 countries and regions, 2008-19: a longitudinal study. Lancet Psychiatry 2021 8 1071 82 10.1016/S2215-0366(21)00292-3 34801129 PMC9766760

[7] OECD Full report: health at a glance. Europe. 2024Available fromhttps://www.oecd.org/en/publications/health-at-a-glance-europe-2024_b3704e14-en/full-report.html Accessed: March 19, 2026

[8] Abbing-Karahagopian V Huerta C Souverein PC de Abajo F Leufkens HGM Slattery J Antidepressant prescribing in five European countries: application of common definitions to assess the prevalence, clinical observations, and methodological implications. Eur J Clin Pharmacol 2014 70 849 57 10.1007/s00228-014-1676-z 24793010

[9] Noordam R Aarts N Verhamme KM Sturkenboom MCM Stricker BH Visser LE Prescription and indication trends of antidepressant drugs in the Netherlands between 1996 and 2012: a dynamic population-based study. Eur J Clin Pharmacol 2015 71 369 75 10.1007/s00228-014-1803-x 25560052

[10] Giovannini S Onder G van der Roest HG Topinkova E Gindin J Cipriani MC Use of antidepressant medications among older adults in European long-term care facilities: a cross-sectional analysis from the SHELTER study. BMC Geriatr 2020 20 310 10.1186/s12877-020-01730-5 32854659 PMC7457305

[11] Forns J Pottegård A Reinders T Poblador-Plou B Morros R Brandt L Antidepressant use in Denmark, Germany, Spain, and Sweden between 2009 and 2014: Incidence and comorbidities of antidepressant initiators. J Affect Disord 2019 249 242 52 10.1016/j.jad.2019.02.010 30780117

[12] Cebron Lipovec N Anderlic A Locatelli I General antidepressants prescribing trends 2009-2018 in Slovenia: a cross-sectional retrospective database study. Int J Psychiatry Clin Pract 2022 26 401 5 10.1080/13651501.2022.2057331 35416749

[13] Stimac D Vukusić I Culig J Sostar Z Bucalić M Outpatient utilization of psychopharmaceuticals: comparison between Croatia and Scandinavian countries (2001-2003). Coll Antropol 2009 33 237 43 19408632

[14] Polić-Vižintin M Stimac D Sostar Z Tripković I Distribution and trends in outpatient utilization of generic versus brand name psychopharmaceuticals during a ten-year period in Croatia. BMC Health Serv Res 2014 14 343 10.1186/1472-6963-14-343 25128190 PMC4261909

[15] Zakon o obveznom zdravstvenom osiguranju - Zakon.hr. Available from: https://www.zakon.hr/z/192/zakon-o-obveznom-zdravstvenom-osiguranju*.* Accessed: March 19, 2026.

[16] eLijekovi. Available from: https://elijekovi-hzzo.gov.hr/. Accessed: March 19, 2026.

[17] von Elm E Altman DG Egger M Pocock SJ Gøtzsche PC Vandenbroucke JP The Strengthening the Reporting of Observational Studies in Epidemiology (STROBE) statement: guidelines for reporting observational studies. J Clin Epidemiol 2008 61 344 9 10.1016/j.jclinepi.2007.11.008 18313558

[18] Anatomical Therapeutic Chemical (ATC) ClassificationAvailable from: https://www.who.int/tools/atc-ddd-toolkit/atc-classification. Accessed: March 19, 2026.

[19] Defined Daily Dose (DDD)Available from: https://www.who.int/tools/atc-ddd-toolkit/about-ddd. Accessed: March 19, 2026.

[20] Državni zavod za statistiku. Population Estimate. Available from: https://podaci.dzs.hr/en/statistics/population/population-estimate/. Accessed: March 19, 2026.

[21] ICD-10 Version 2019. Available from: https://icd.who.int/browse10/2019/en. Accessed: March 19, 2026.

[22] Lalji HM McGrogan A Bailey SJ An analysis of antidepressant prescribing trends in England 2015-2019. J Affect Disord Rep 2021 6 100205 10.1016/j.jadr.2021.100205 34957433 PMC8684293

[23] Wallis KA, Dikken PJS, Sooriyaarachchi P, Bohnen AM, Donald M. Lessons from the Netherlands for Australia: cross-country comparison of trends in antidepressant dispensing 2013-2021 and contextual factors influencing prescribing. Aust J Prim Health. 2024 Feb;30(1):NULL. 10.1071/PY23168 38056885

[24] OECD 2023. Pharmaceutical consumption: Health at a Glance 2023. Available from: https://www.oecd.org/en/publications/health-at-a-glance-2023_7a7afb35-en/full-report/pharmaceutical-consumption_4b6cb013.html. Accessed: March 19, 2026.

[25] Selke Krulichová I Hallberg A Selke GW Aaltonen K Casula M Fürst J Impact of the COVID-19 pandemic on antidepressant use in eleven European regions: a comparative time series analysis 2018-2022. Soc Psychiatry Psychiatr Epidemiol 2025 40696201 10.1007/s00127-025-02962-9 PMC13021844

[26] Tiger M Wesselhoeft R Karlsson P Handal M Bliddal M Cesta CE Utilization of antidepressants, anxiolytics, and hypnotics during the COVID-19 pandemic in Scandinavia. J Affect Disord 2023 323 292 8 10.1016/j.jad.2022.11.068 36442654 PMC9691511

[27] De Bandt D Haile SR Devillers L Bourrion B Menges D Prescriptions of antidepressants and anxiolytics in France 2012-2022 and changes with the COVID-19 pandemic: interrupted time series analysis. BMJ Ment Health 2024 27 e301026 10.1136/bmjment-2024-301026 38413052 PMC10900346

[28] Maguire A Kent L O’Neill S O’Hagan D O’Reilly D Impact of the COVID-19 pandemic on psychotropic medication uptake: time-series analysis of a population-wide cohort. Br J Psychiatry J Ment Sci. 2022 221 748 57 10.1192/bjp.2022.112 35968915

[29] Lee TC Vigod S Bortolussi-Courval É Hanula R Boulware DR Lenze EJ Fluvoxamine for outpatient management of COVID-19 to Prevent hospitalization: a systematic review and meta-analysis. JAMA Netw Open 2022 5 e226269 10.1001/jamanetworkopen.2022.6269 35385087 PMC8987902

[30] Vukićević T Draganić P Škribulja M Puljak L Došenović S Consumption of psychotropic drugs in Croatia before and during the COVID-19 pandemic: a 10-year longitudinal study (2012-2021). Soc Psychiatry Psychiatr Epidemiol 2024 59 799 811 10.1007/s00127-023-02574-1 37847256

[31] Ishtiak-Ahmed K Köhler-Forsberg O Mortensen EL Nierenberg AA Gasse C trends, patterns and associated user characteristics of antidepressant prescriptions in older adults: a nationwide descriptive cohort study in Denmark. Drugs Aging 2023 40 355 68 10.1007/s40266-023-01018-4 36920735

[32] Montgomery S Hansen T Kasper S Efficacy of escitalopram compared to citalopram: a meta-analysis. Int J Neuropsychopharmacol 2011 14 261 8 10.1017/S146114571000115X 20875220

[33] PSUSA/00002943/201806 - periodic safety update report single assessment | European Medicines Agency (EMA). 2023. Available from: https://www.ema.europa.eu/en/medicines/psusa/psusa-00002943-201806*.* Accessed: March 23, 2026.

[34] Commissioner O of the. Tianeptine Products Linked to Serious Harm, Overdoses, Death. FDA. 2025 May 9]. Available from: https://www.fda.gov/consumers/consumer-updates/tianeptine-products-linked-serious-harm-overdoses-death. Accessed: March 23, 2026.

[35] Patel K Allen S Haque MN Angelescu I Baumeister D Tracy DK Bupropion: a systematic review and meta-analysis of effectiveness as an antidepressant. Ther Adv Psychopharmacol 2016 6 99 144 10.1177/2045125316629071 27141292 PMC4837968

[36] Jurcevic A, Raic L, Buble T, Svajda M. Rising antidepressant use among youth in Croatia (2016–2023): a widening gap between the sexes. Eur J Public Health. 2025 Oct 27;35(Suppl 4):ckaf161.1004.

[37] Selke Krulichová I Hallberg A Selke GW Aaltonen K Casula M Fürst J Impact of the COVID-19 pandemic on antidepressant use in eleven European regions: a comparative time series analysis 2018-2022. Soc Psychiatry Psychiatr Epidemiol 2025 40696201 10.1007/s00127-025-02962-9 PMC13021844

[38] Fassmer AM Wandscher K Bedri A Jobski K Poustka L Bachmann CJ Change of antidepressant utilization in children, adolescents and young adults in Europe before and during the COVID-19 pandemic: a systematic review. Eur Child Adolesc Psychiatry 2026 35 3 16 10.1007/s00787-025-02839-x 40810963 PMC12916912

[39] Recommendations | Depression in children and young people: identification and management | Guidance | NICE. 2019. Available from: https://www.nice.org.uk/guidance/ng134/chapter/Recommendations. Accessed: March 23, 2026.

[40] European Medicines Agency finalises review of antidepressants in children and adolescents | European Medicines Agency (EMA). 2005. Available from: https://www.ema.europa.eu/en/news/european-medicines-agency-finalises-review-antidepressants-children-adolescents. Accessed: March 23, 2026.

[41] Hetrick SE McKenzie JE Bailey AP Sharma V Moller CI Badcock PB New generation antidepressants for depression in children and adolescents: a network meta-analysis. Cochrane Database Syst Rev 2021 5 CD013674 34029378 10.1002/14651858.CD013674.pub2 PMC8143444

[42] Prozac - referral | European Medicines Agency (EMA). 2006. Available from: https://www.ema.europa.eu/en/medicines/human/referrals/prozac. Accessed: March 23, 2026.

[43] Zoloft - referral | European Medicines Agency (EMA). 2009. Available from: https://www.ema.europa.eu/en/medicines/human/referrals/zoloft. Accessed: March 23, 2026.

[44] HALMED. Fevarin 100 mg filmom obložene tablete - Baza lijekova | Lijekovi. HALMED. Available from: https://www.halmed.hr/Lijekovi/Baza-lijekova/Fevarin-100-mg-filmom-oblozene-tablete/13514/. Accessed: March 23, 2026.

