# Supplementary material for: Antidepressant prescribing pattern in Croatia: a retrospective, longitudinal study from 2017 to 2022
Source: Croat Med J. 2026 Apr;67(2):55–65. doi: 10.3325/cmj.2026.67.55 (PMC13176931; doi:10.3325/cmj.2026.67.55)

Supplemental Figure 1. Trend in utilisation for individual antidepressants prescribed in Croatia in period 2017-2022.

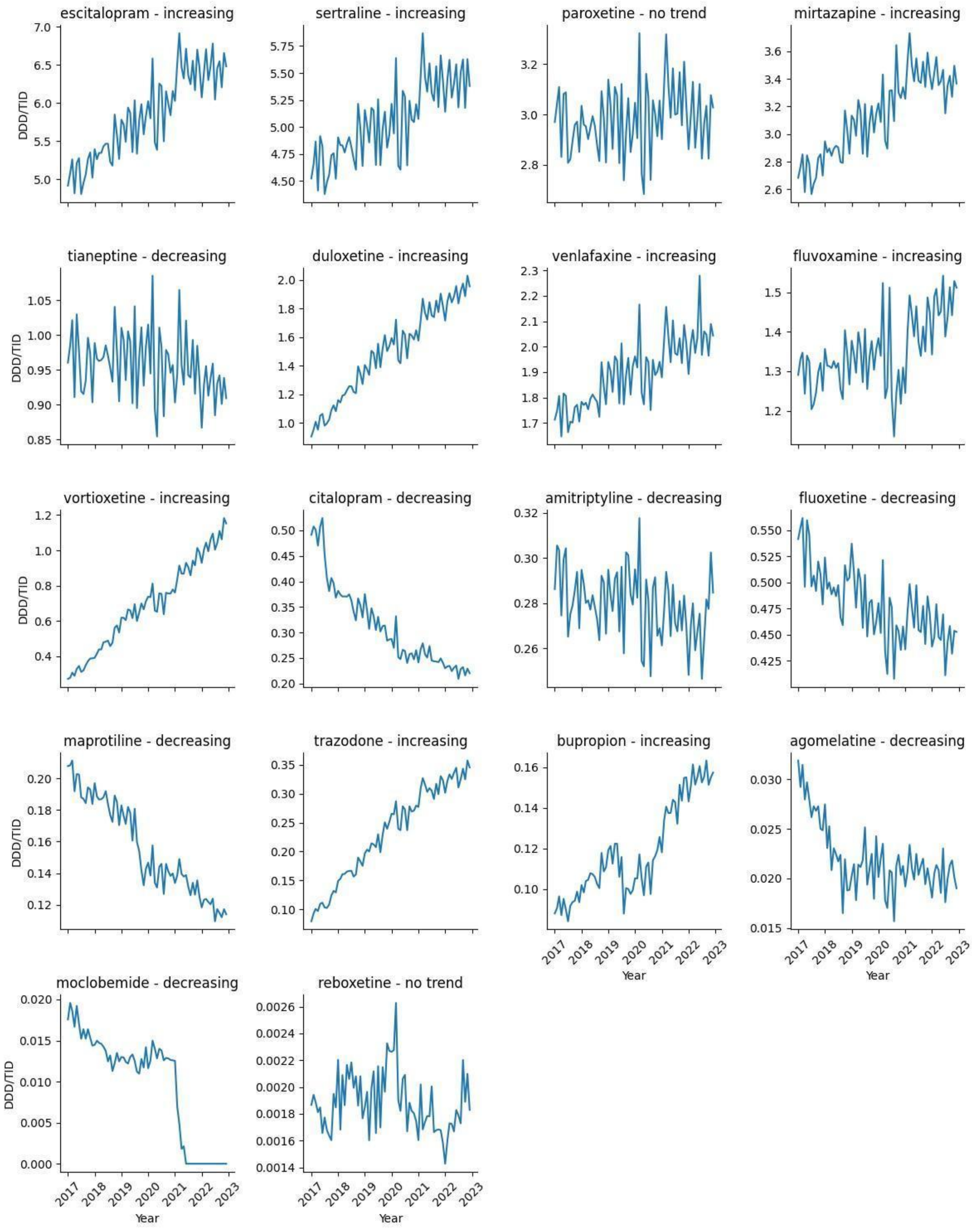

Supplement: Supplementary Figure 1 [file CroatMedJ_67_s001.pdf]
