# Supplementary material for: Antidepressant prescribing pattern in Croatia: a retrospective, longitudinal study from 2017 to 2022
Source: Croat Med J. 2026 Apr;67(2):55–65. doi: 10.3325/cmj.2026.67.55 (PMC13176931; doi:10.3325/cmj.2026.67.55)
Supplement: Supplementary Table 1 [file CroatMedJ_67_s002.pdf]

Supplemental Table 1. Cross-tabulated gender and age group prescription analysis (F – females; M – males; Rx – prescriptions). Data are presented as the number of prescriptions and prescription rates per 1,000 persons per year for females and males within each age group, together with crude and adjusted female-to-male (F/M) prescription ratios.

| Age group | F (n) | M (n) | Raw F/M ratio | F rate (Rx/1000/yr) | M rate (Rx/1000/yr) | Adjusted F/M ratio |
|---|---|---|---|---|---|---|
| 0–17 | 36833 | 18260 | 2,02 | 18,3 | 8,6 | 2,13 |
| 18–25 | 118584 | 78106 | 1,52 | 115 | 71 | 1,62 |
| 26–44 | 833203 | 576250 | 1,45 | 289,5 | 192,2 | 1,51 |
| 45–64 | 2321720 | 1612702 | 1,44 | 676,9 | 491,2 | 1,38 |
| 65+ | 2237536 | 827746 | 2,7 | 740,4 | 393,3 | 1,88 |
